# Gene expression analysis of whole blood RNA from pigs infected with low and high pathogenic African swine fever viruses

**DOI:** 10.1038/s41598-017-10186-4

**Published:** 2017-08-31

**Authors:** Crystal Jaing, Raymond R. R. Rowland, Jonathan E. Allen, Andrea Certoma, James B. Thissen, John Bingham, Brenton Rowe, John R. White, James W. Wynne, Dayna Johnson, Natasha N. Gaudreault, David T. Williams

**Affiliations:** 1 Physical & Life Sciences Directorate, Lawrence Livermore National Laboratory, Livermore, California, United States of America; 2 Department of Diagnostic Medicine and Pathobiology, Kansas State University, Manhattan, Kansas United States of America; 3 Computation Directorate, Lawrence Livermore National Laboratory, Livermore, California, United States of America; 4 CSIRO Australian Animal Health Laboratory, Geelong, Victoria Australia

## Abstract

African swine fever virus (ASFV) is a macrophage-tropic virus responsible for ASF, a transboundary disease that threatens swine production world-wide. Since there are no vaccines available to control ASF after an outbreak, obtaining an understanding of the virus-host interaction is important for developing new intervention strategies. In this study, a whole transcriptomic RNA-Seq method was used to characterize differentially expressed genes in pigs infected with a low pathogenic ASFV isolate, OUR T88/3 (OURT), or the highly pathogenic Georgia 2007/1 (GRG). After infection, pigs infected with OURT showed no or few clinical signs; whereas, GRG produced clinical signs consistent with acute ASF. RNA-Seq detected the expression of ASFV genes from the whole blood of the GRG, but not the OURT pigs, consistent with the pathotypes of these strains and the replication of GRG in circulating monocytes. Even though GRG and OURT possess different pathogenic properties, there was significant overlap in the most upregulated host genes. A small number of differentially expressed microRNAs were also detected in GRG and OURT pigs. These data confirm previous studies describing the response of macrophages and lymphocytes to ASFV infection, as well as reveal unique gene pathways upregulated in response to infection with GRG.

## Introduction

African swine fever (ASF) is a highly pathogenic transboundary disease of domestic and wild pigs caused by the ASF virus (ASFV). ASFV is endemic in parts of Africa, where it exists in a complex transmission cycle involving soft ticks, as well as direct pig-to-pig transmission^1^. After being eradicated from mainland Europe in 1995, ASF re-emerged in the Caucasus in 2007 and has subsequently spread to domestic pigs and wild boar in Europe. There are no effective treatments or vaccines available so disease control is based on the enforcement of strict quarantine and stamping out measures^2, 3^. Impacts of ASFV infection include increased mortality and morbidity, loss of trade, and the costs associated with outbreak response and eradication measures. The presence and spread of ASFV in Europe and continued transmission in Africa makes the threat of ASF a global concern.

ASFV is an enveloped virus that contains a 170–190 kb double stranded DNA genome encoding 150–167 genes. It is the only member of the family *Asfarviridae*, genus *Asfivirus*. Viral proteins participate in nucleotide metabolism, transcription, DNA replication and repair, virion structure, morphogenesis and the modulation of host immunity^4^. Several open reading frames code for genes of unknown function. The virion is composed of between 30 and 50 polypeptides. Among them, 21 have been identified as structural proteins^5^. Virulence of virus strains ranges from highly pathogenic, causing death within a few days, to low pathogenic, causing sub-clinical or persistent infection with low levels of morbidity and mortality. The virus primarily targets cells of the mononuclear phagocyte system, including monocytes that circulate in the blood^6–9^. It is the unique interaction between ASFV and its host macrophage that determine the pathogenic outcome.

The purpose of this study was to compare both host and viral gene expression responses in circulating cell populations during infection with high and low virulent isolates. The low pathogenic genotype 1 virus, OUR T88/3 (OURT), was originally isolated from *Ornithodoros erraticus* in Portugal and is characterized as causing no apparent clinical signs^10^. The attenuated phenotype is a property that makes OURT a good modified live virus vaccine candidate^11^. In contrast, GRG2007/1 (GRG) is a highly pathogenic genotype 2 virus, first isolated in Georgia in 2007^12^. Infection of domestic pigs with GRG results in severe disease characterized by a hemorrhagic fever and high rates of mortality^13^. In this study, we applied RNA-Seq to analyze gene expression in whole blood from pigs following infection with OURT or GRG. The results show both common and unique gene expression patterns between OURT and GRG, including the upregulated expression of host genes associated with macrophages and NK cells, and viral genes associated with modification of host immunity.

## Results

### Clinical outcome and pathology

**Figure 1:**
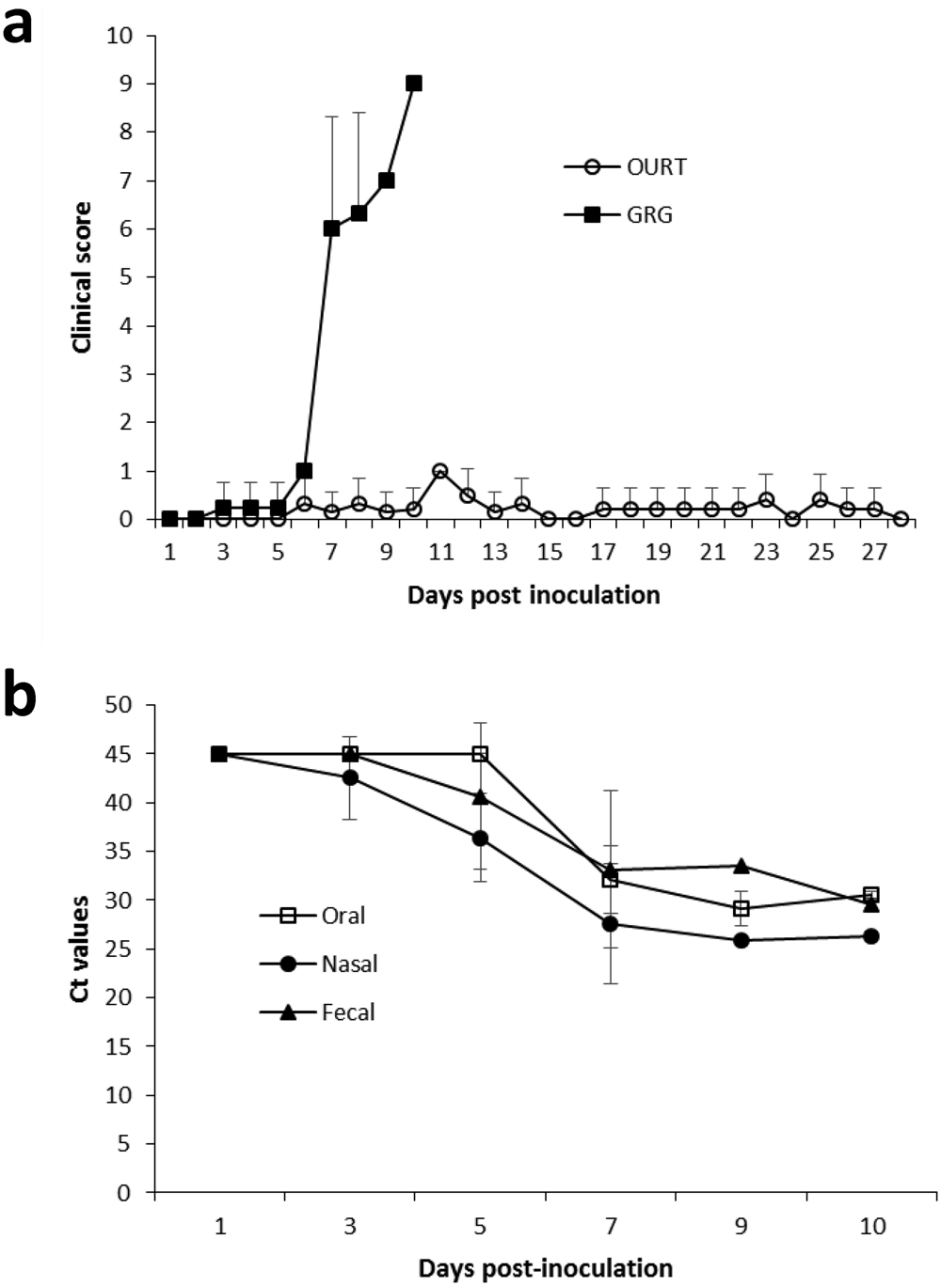
Clinical scores for GRG- and OURT-infected pigs, and virus shedding for GRG-infected pigs. Panel a shows clinical scores for GRG (solid squares) and OURT (open circles) pigs. The results show the mean and standard deviation for six pigs infected with OURT and four pigs infected with GRG. The GRG pigs were terminated between 7 and 10 days after infection. Panel b shows virus shedding in the GRG pigs. Shedding was determined by measuring virus in oral, nasal and fecal samples. Viral nucleic acid was not detected in samples from the OURT pigs.

Back titration of the viral inocula indicated that OURT pigs received approximately 10^6^ TCID_50_, as intended, but GRG pigs received 10^4^ TCID_50_. Clinical scores for pigs following inoculation with ASFV are summarized in Fig. 1a. Clinical scores for individual pigs are shown in Supplementary Fig. S1. For the OURT pigs, only mild or non-specific clinical signs were observed, with skin lesions being the most common. Lesions occurred most commonly on the back (6/6 pigs) and were also found on the ears (2/6), shoulder (1/6), leg (1/6), and over the eyes (2/6). One pig (number 10) was euthanized on day 14 post-infection (PI), after presenting with reduced body condition and a swollen hind leg with lameness. No clinical signs, lesions or gross pathology were observed in the remaining pigs at day 28, the end of the study. In sharp contrast, pigs inoculated with GRG showed signs and lesions characteristic of acute infection with a highly pathogenic ASFV strain. Clinical scores were observed from day 3 PI and rapidly increased after day 6 PI (Fig. 1a) and included fever (>41 °C), skin lesions, diarrhea, conjunctivitis and decreased activity. Other ASF-associated clinical signs were apparent, including anorexia, reduced body condition, swollen joints and lameness, dyspnea, bloody diarrhea and cyanosis. All pigs met pre-determined humane endpoints and were euthanized between 7–10 days PI. Postmortem examination of organs showed hemorrhagic lymph nodes, accumulation of fluid in the abdominal, thoracic and pericardial cavities, multifocal sub-capsular petechiae on kidneys, and severe distention of the wall of the gall bladder with blood. At the microscopic level, mild to severe edema and congestion were present in the lungs. Focal necrosis was present in spleen, lymph nodes, and tonsil. The liver showed congestion with periacinal necrosis. ASFV antigen was detected in all tissues examined with a predominance of staining in macrophages associated with lymphoid and parenchymal tissues (Supplementary Fig. S2). Viral antigen was also found in occasional hepatocytes as diffuse cytoplasmic staining (p30 and p72) and as large, usually single intra-cytoplasmic inclusion bodies (p72) and in single cells of the tonsillar crypt epithelium. Because of an absence of gross anatomical changes, histopathological examination was not performed on the OURT pigs.

Prior to inoculation with ASFV, blood samples were collected for diagnostic testing of common swine viral pathogens. Notably, two pigs (numbers 10, 11) in the OURT group were positive for porcine circovirus type 2 (PCV2) DNA and antibodies. Subsequently, PCV2 was detected by PCR in the remaining pigs at day 7 (numbers 8, 9) or day 14 (numbers 7, 12) and all but one pig (number 12) was seropositive for PCV2 at the end of the experiment (day 28 PI). Histological testing of lymphoid tissues taken at necropsy showed no significant lesions and no PCV2 antigen was detected by immunohistochemistry. From these results, together with the observation of either no or mild clinical signs (≤1; Fig. 1a, Supplementary Fig. S1) and steady weight gain (results not shown), we found no indication of PCV2-associated disease (PCVAD). There was no evidence of PCV2 infection of pigs in the GRG group.

### Serology, virus shedding and virus load

By 28 days PI, four of six pigs inoculated with OURT seroconverted, with serum indirect fluorescent antibody (IFA) titers ranging from 160 (pigs 7, 10, 11) to 2560 (pig 12). ASFV PCR performed on swabs, whole blood and tissues showed only one of the six pigs infected with OURT as weakly positive for ASFV nucleic acid in spleen and submandibular lymph node (pig 10 at 14 days). These results are consistent with previous reports of pig infections with OURT^10, 14^. Together these data showed that four of the OURT pigs were productively infected, but at a low level. Of the GRG-infected pigs, none were antibody positive, consistent with the early acute phase of the disease. Virus shedding was apparent by the presence of nucleic acid in nasal swabs beginning at day 3, fecal swabs from day 5 and in oral swabs beginning at day 7 (Fig. 1b; Supplementary Table S1). A summary of PCR and virus isolation results from testing tissues and blood is shown in Supplementary Table S2. Highest viral titers were found in the blood, ranging between 9.5 and 10.0 TCID_50_/mL, followed by the spleen (8.0 to 8.5 TCID_50_/mL) and lymph nodes (5.2 to 8.8 TCID_50_/mL). The large quantity of virus present in blood and tissues is consistent with the highly pathogenic nature of GRG.

### Sequencing and gene expression statistics

Three of the four OURT- or GRG-infected pigs in each group were randomly selected for sequencing of total RNA isolated from whole blood. In the OURT group, pigs 7, 11 and 12 were selected, while in the GRG group, pigs 13, 14 and 16 were selected. The number of sequencing reads obtained from each sample is summarized in Supplementary Table S3. An average of 43.2 M reads were obtained for the OURT pig samples (range 36.8 M to 58.1 M), and 37.8 M reads for the GRG samples (range 26.6 M to 39.1 M). All mapped reads were used to estimate gene expression; however, reads mapping to multiple genes contributed only partial expression counts. The results for the most significant differentially regulated genes (DEGs) from the OURT and GRG pigs are summarized in Supplementary Table S4.

Expression levels greater than 2 log_2_ were considered as “highly differentially expressed”. The GRG expression data were obtained from 3 pigs (numbers 13, 14 and 16) euthanized at 7, 8 and 10 days after infection. The gene expression results were averaged for the three pigs (treated as biological replicates) using cufflinks to identify genes with significant differential expression. Within the group of upregulated genes, 76 of the 217 genes were highly upregulated. Within the group of 178 downregulated genes, 110 were highly downregulated. In general, there were lower numbers of DEGs for the OURT pigs (numbers 7, 11 and 12) over the duration of the infection trial. The exception was the D0 vs D28 data, which showed a total of 490 DEGs. However, only 22 DEGs were highly expressed.

### ASFV gene expression

Since ASFV replicates in circulating monocytes, ASFV mRNA was detected in whole blood RNA. ASFV gene reads were found in all blood samples analyzed from GRG pigs at termination, but in none of the OURT samples, consistent with the absence of detectable quantities of OURT nucleic acid in the blood. The number of ASFV sequence reads mapping to GRG genes constituted about 0.1% of the overall reads. RNA-Seq detected the expression of 109 named ASFV genes and multi-gene families (MGFs). A complete list of ASFV genes detected by RNA-Seq is found in Supplementary Table S5. To ensure the RNA transcripts detected are from the mRNA of the gene sequence and not “read through” from upstream genes, the genes that show at least a two fold increase in gene expression relative to their neighbor genes in at least two pigs are selected as the top expressed ASFV genes (Table 1). As shown in the table, there was frequently a wide variation in Fragments Per Kilobase per Million mapped fragments (FPKM) values observed between pigs. The reason for this difference is not clear, but could be related to the time each pig was sacrificed, or the relative number of susceptible macrophages. Within the group of ASFV-expressed genes were structural, non-structural and host regulatory genes. A fourth group included genes with no known function. The highest mean level of expression was found for *MGF 360–15R* (*A276R*), which codes for an early gene, whose function blocks early innate responses by inhibiting the induction of *IFN-β* ^15^. Another highly expressed gene, *DP71L*, is also involved in the subversion of innate immunity. The importance of *DP71L* and upregulation of the host cell homolog, *PPP1R15A*, in the regulation of host cell translation is discussed in more detail later. Major structural genes detected included *CP204L*, which codes for p30, an immunodominant phosphoprotein of the virion and a preferential target for serological detection of infection. Additional genes that code for the proteins E184L, CP204L, CP312R, K205R, K145R, and K196R form a group of 12 proteins identified as the most immunodominant antigens produced during infection^16^. Therefore, the immunodominant nature of these six proteins can be explained by relatively high levels of cognate gene expression.

**Table 1:** Top 17 ASFV genes detected in GRG-infected pigs^a^.

Gene	FPKM				Description	Reference
	Pig 13 Day 8	Pig 14 Day 7	Pig 16 Day 10	Mean		
*MGF 360-15R*	1,190,000	ND^b^	19,979	403,326	A276R inhibits induction of IFN-beta	15
*E184L*	239,572	245,242	158,848	214,554	Immunodominant protein	16
*DP71L*	261,200	ND	267,893	176,364	Cofactor for host PP1c to dephosphorylate P-eIF2a	24
*CP204L*	84,132	82,778	148,830	105,247	Immunodominant, p30 structural protein	16, 55
*MGF 360-1L*	238,149	15,569	2,557	85,425	KP360L, early gene	56
*CP312R*	116,113	ND	111,889	76,000	Immunodominant protein	16
*K205R*	68,096	58,253	50,243	58,864	Early immunodominant protein associated with viral factories	16, 57
*A137R*	128,520	47,590	ND	58,703	P11.5 structural protein	58
*I73R*	98,209	77,236	ND	58,481	Contains a tandem repeat sequence	59
*DP238L*	103,860	25,502	45,057	58,139	Pre-replicative gene	60
*E120R*	27,619	83,321	52,669	54,536	DNA binding protein	61
*MGF 110-4L*	ND	103,196	44,404	49,200	XP124L, KDEL-like domain	62
*MGF 110-7L*	27,087	34,233	34,432	31,917	Unassigned	
*K196R, K421R, K78R*	61,278	25,421	7,228	31,309	K146R, immunodominant, thymidine kinase; K421R and K78R, late genes	16, 63 and 64
*K145R*	52,927	31,137	ND	28,021	Unassigned	
*I215L*	ND	31,817	45,810	25,876	Ubiquitin conjugating enzyme	59
*L83L*	32,244	ND	42,655	24,966	Unassigned	

^a^Genes selected based on mean FPKM for three pigs.

^b^ND, not detected.

### DEGs upregulated in GRG pigs

The DEGs for GRG pigs are listed in Supplementary Table S6. Table 2 summarizes the top 20 upregulated genes in the GRG pigs. When taken together, 17 of the 20 top-upregulated host genes could be directly or indirectly associated with the response to infection with a highly pathogenic ASFV isolate. In general, these genes could be divided into four groups: 1) genes associated with macrophages, 2) genes linked with virus infection, including ASFV infection, 3) lymphocyte-associated genes with an emphasis on NK cells, and 4) genes not associated with virus infection or immunity. It is interesting to note that two of the genes in the last category, *TGM3* and *GPATCH4*, are associated with autoimmunity^17, 18^.

**Table 2:** The top 20 upregulated genes in pigs infected with GRG.

Gene	Log_2_	Gene Product	Function	Ref.
**Genes associated with monocyte macrophages**				
*S100A8*	4.8	S100A8	Bind CD68-Regulates activation	65
*S100A9*	4.5	S100A9	Bind CD68-Regulates activation	65
*SIGLEC-1*	3.3	Sialoadhesin, CD169	Surface protein that binds sialic acid	66
*HMOX1*	3.8	Hemeoxigenase-1	Heme metabolism in CD163 macrophages	67
*RGS1*	3.4	RGS-1	Desensitization of chemoattractant signaling	68
*CCL5*	3.9	Chemokine CCL5	Upregulated in ASFV-infected macrophages	69
**Genes associated with virus (ASFV) infection**				
*IDO1*	3.8	Indoleamine 2,3-dioxygenase 1	Upregulated by interferon after virus infection	70
*HSP70.2*	4.2	Heat Shock Protein 70	Upregulated in ASFV-infected Vero cells	71
*PPP1R15A*	4.0	GADD34	Directs the dephosphorylation of eIF2a by PP1	72
*XKR8*	3.7	Xkr8	Facilitates apoptosis by increasing surface exposure of phosphatidylserine	73
*ABCA1*	3.4	ATP-binding cassette transporter A1	Regulates cholesterol metabolism during virus infection	74
*RTF1*	3.7	Rtf1	Regulates gene transcription, associated with adenovirus replication	75
miR-122	7.5	miRNA-122	Increase associated with liver damage during HCV infection	20
**Genes associated with lymphocytes (T cells and/or NK cells)**				
*NKL*	5.3	NK-lysin	Antimicrobial peptide	76
*BATF*	4.2	BATF	Transcription factor in B and T cells	77
*GZMA*	4.0	Granzyme A	Proapoptotic serine protease	78
*PRF1*	4.0	Perforin	Cytolytic protein for cells and bacteria	79
**Other genes**				
*LTF*	5.6	Lactotransferrin	Antimicrobial peptide found in neutrophils	80
*TGM3*	3.9	Transglutaminase 3	Forms covalent bond between proteins	17
*GPATCH4*	3.5	GPATCH4	DNA/RNA binding protein, autoantigen	18

In terms of genes associated with macrophages, the upregulation of the macrophage surface marker gene, *SIGLEC-1* (increased by a factor of 3.3 log_2_; Supplementary Table S6), which codes for sialoadhesin or CD169, suggests that increased *SIGLEC-1* expression relates to increased numbers of circulating monocytes. The upregulation of a second macrophage-associated marker gene, *CD163* (increased by a factor of 2.9 log_2_) further supports increased numbers of macrophages. Linked with increased *CD163* expression was the upregulation of *HMOX1* (increased by a factor of 3.82 log_2_), which codes for hemeoxigenase-1, a protein found in *CD163*-positive macrophages. Other upregulated macrophage-associated genes included *S100A8/A9*. A second group of macrophage-associated genes included several chemokines and chemokine receptors (Supplementary Table S6), which included *CCL5*, previously described as upregulated during acute ASFV infection^19^. The second set of upregulated genes was grouped according to the response of cells or tissues to virus infection, including host genes that block virus infection or subvert host cell responses, as well as genes that may facilitate virus replication. An example of the later is *PPP1R15A*, which codes for GADD34, a host protein involved in the dephosphorylation of P-eIF2α. As described in more detail below, the expression of the ASFV homolog, *DP71L* (Table 1), identifies a redundant mechanism to ensure that host cell translation remains intact. The remaining upregulated genes associated with ASFV infection are likely involved in the innate response of macrophages to virus infection. The most highly upregulated gene was associated with the microRNA (miRNA) miR-122, which is linked to the replication of hepatitis C virus (HCV). In response to liver damage, circulating levels of miR-122 are increased^20^. The presence of increased miR-122 could be the result of liver damage caused by the ASFV infection of liver macrophages. Interestingly, miR-122 was the only miRNA detected during GRG infection. Several miRNAs were upregulated during OURT infection, including miR-328, miR-181, miR-1296 and miR-199 (Supplementary Table S6). The third group of genes upregulated in response to GRG infection was associated with lymphocyte function. The 7 to 10 days’ time frame covered by the collection of the GRG samples likely reflects early responses to infection, such as the activation of NK cells along with the transition of the host to adaptive immunity through the activation and expansion of B and T cells. Upregulated NK cell genes such as *NKL*, *PRF1*, and *GZMA*, are associated with the killing of infected target cells.

To compare with the GRG RNA-Seq data, we evaluated the top 20 upregulated genes at 7 days after infection with OURT (Table 3). The top 20 upregulated genes could be placed into similar groups identified for GRG pigs (Table 3). However, the largest category consisted of upregulated genes described as “other” or not directly linked to infection. Several genes in this category could be linked with normal developmental and metabolic processes. Genes associated with the response of macrophages to infection included macrophage-associated chemokine genes, *CX3R1* and *CCL5*. Overlap between the GRG and OURT groups were found in genes associated with NK and T cell functions. For example, *GZMA* and *NKL*, upregulated in the OURT pigs were also upregulated in the GRG pigs.

**Table 3:** Top 20 upregulated DEGs from day 7 in pigs infected with OURT.

Gene	Log_2_	Product	Function	Ref.
**Genes associated with monocyte macrophages**				
*PLA2G6*	7.1	Cytosolic phospholipase A2a	Production of bioactive lipids	81
*MIR328*	5.7	miR-328	Regulation of bacterial killing	82
*CX3CR1*	2.6	CX3C Chemokine receptor	Receptor associated with wound repair	83
CCL5*	1.7	Chemokine CCL5	Upregulated in ASFV-infected macrophages	69
**NK/T cell-associated genes**				
*KLRK1*	3.0	NKG2D	Receptor for cell-mediated cytoxicity	84
*GPR56*	2.3	GPR56	Expressed on the surface of cytotoxic NK and T cells	85
GZMA*	1.9	Granzyme A	Pro-apoptotic serine protease	78
NKL*	1.9	NK-lysin	Antimicrobial peptide	76
*FASLG*	1.9	FAS ligand	Pro-apoptotic protein	86
*SEMA6D*	3.8	Semaphorin 6D	Regulates T cell function	87
**Infection-associated genes**				
RTF1*	3.7	Rtf1	Regulation of gene transcription associated with adenovirus replication	75
**Other genes**				
*SYTL2*	2.0	Synaptotagmin-like protein 2	Epithelial cell development	88
*CENPW*	2.4	CENP-W	Centromere protein	89
*DRK1*	2.7	delayed rectifier potassium channel 1 (DRK1)	Potassium channel protein	90
*SAMD3*	2.7	Samd3	Cell proliferation and development	91
*COL5A2*	3.2	Type V collagen	Skeletal development	92
*MYH1*	3.5	Myosin heavy chain	Muscle fiber development in pigs	93
*PTH1R*	4.3	Parathyroid hormone receptor	Bone and cartilage metabolism and kidney function	94
*SULT1E1*	5.4	Estrogen sulfotransferase	Estrogen metabolism	95
*GGH*	4.1	Gamma-glutamyl hydrolase	Participates in glutathione metabolism	96

*Also upregulated in GRG-infected pigs (Table 3).

The top downregulated genes were also evaluated. Unlike the genes upregulated in response to ASFV infection, the top downregulated genes for the GRG and OURT pigs could not be linked with any category associated with virus infection, macrophage function, or the activation of the immune system (Supplementary Table S6). This observation was surprising since RNA-Seq showed that several of these genes were highly downregulated with reduced expression as low as −13.1 log_2_ (Supplementary Table S6).

### Mapping genes to KEGG pathways

The pathways with the most significant number of genes represented were identified using the DAVID enrichment tool (Table 4 and Supplementary Table S7). Even though GRG pigs possessed the greatest number of DEGs, the KEGG pathways for the OURT pigs showed the greatest number of DEGs that could be mapped to a specific pathway. The three pathways with the greatest number of DEGs were linked to immune-related functions. The three pathways contained several genes described as highly upregulated. For example, pathway hsa04060, cytokine-cytokine receptor interaction, possessed three highly upregulated genes, including *FASLG* (Fas ligand transcript variant 1), *IL2RB* (Interleukin 2 receptor subunit beta) and *CX3CR1* (C-X3-C motif chemokine receptor 1). Another pathway with three mapped upregulated genes was hsa04650, natural killer cell-mediated cytotoxicity, comprising the genes *PRF1* (perforin 1), *FASLG* and *KLRK1* (killer cell lectin like receptor K1). These data provide further support for upregulated NK cell activity during ASFV infection. For GRG, only one pathway was identified as significant: hsa04622, the RIG-I-like receptor (RLR) signaling pathway. The three upregulated genes in this pathway were: *DHX58* (DEXH-box helicase 58), *CXCL10* (C-X-C motif chemokine 10) and *NFKBIA* (NFKB inhibitor alpha).

**Table 4:** KEGG pathways with a significant number of DEGs.

Virus	Pathway	Description	P Value^a^	Genes^b^
OURT	hsa04060	Cytokine-cytokine receptor interaction	0.091	*CX3CR1*, *IL2RB*, *FASLG*
	hsa04650	NK cell-mediated cytotoxicity	0.027	*PRF1*, *KLRK1*, *FASLG*
GRG	hsa04622	RIG-I-like receptor signaling pathway	0.077	*DHX58*, *CXCL10, NFKBIA*

^a^P values were calculated using Fisher exact test based on the fraction of genes that map to a specific pathway as compared to the number of background genes associated with the pathways.

^b^Underlined genes are identified as highly upregulated (>2 log_2_; see Supplementary Table S6, Tables 2 and 3).

### DEGs shared by both GRG and OURT pigs

**Figure 2:**
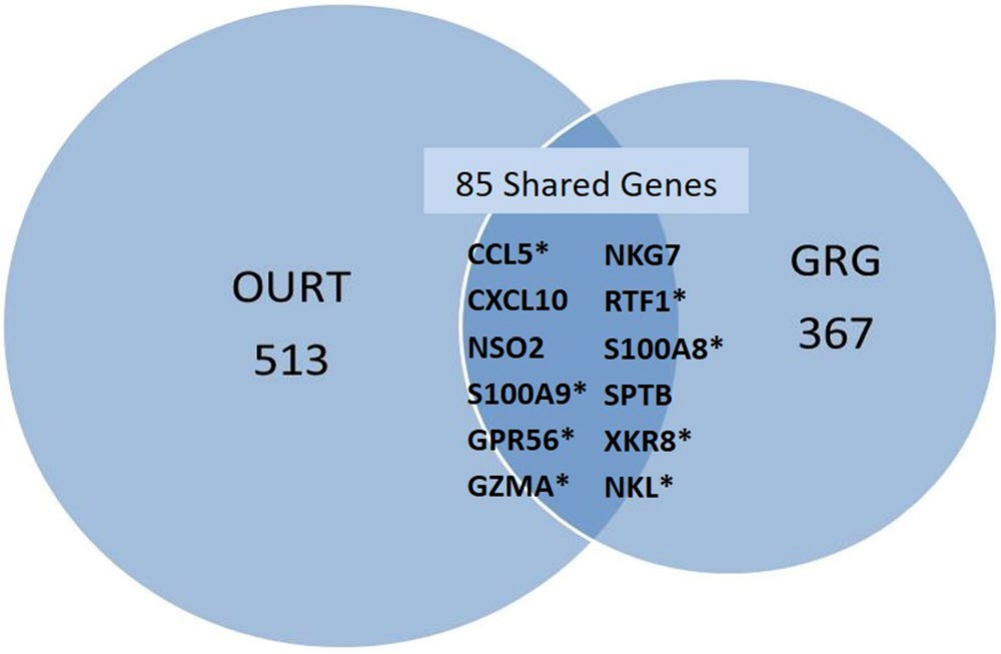
Overlap of DEGs in GRG and OURT pig samples. The Venn diagram shows the overlap of gene expression between GRG and OURT samples for all days post-infection (all samples). The complete list of shared genes is found in Supplementary Table S8. The 12 most highly upregulated genes within the shared group are listed. Genes with an asterisk are further described in Tables 2 or 3.

The principal differences between OURT and GRG pigs were the presence of active ASFV replication in circulating cells along with the enhanced virulence of the GRG isolate. However, both groups shared several genes upregulated in response to infection. A diagrammatic summary of the overlap between total DEGs is presented as a Venn diagram in Fig. 2. Supplementary Table S8 contains a complete list of overlapping DEGs. Of the 880 DEGs for all samples, 85 or about 10% were shared between OURT and GRG samples. The percentages of shared DEGs for the OURT and GRG samples were 17% and 23%, respectively. Twelve genes within the shared group were identified as highly upregulated, of which eight genes (*CCL5*, *S100A8*, *GPR56*, *GZMA*, *RTF1*, *S100A9*, *XKR8*, and *NKL*) are listed in Tables 2 and 3. Most of the 12 highly upregulated genes were associated with macrophages and NK cells.

**Figure 3:**
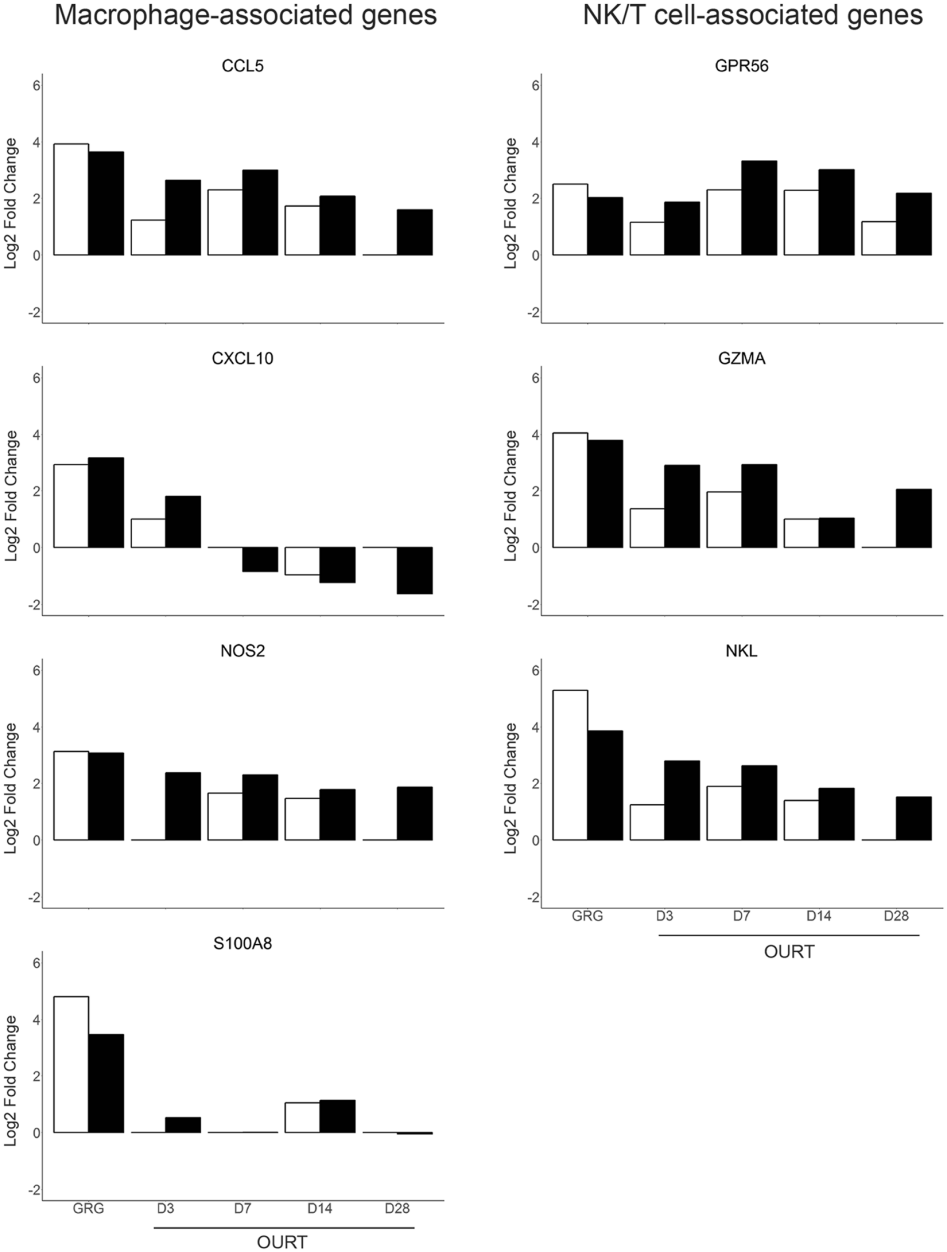
PCR confirmation of gene expression levels for macrophage and NK/T cell-associated genes common to GRG and OURT infection. The closed bars represent the RNA-Seq data. The open bars show the results for qPCR. The expression of β2-microglobulin was used to calculate the expression level measured by qPCR. The results are shown as mean for the individual pigs.

To further confirm the RNA-Seq results, qPCR was performed on seven selected macrophage and NK/T cell-associated genes listed in Fig. 3. In general, the results showed agreement between the qPCR and RNA-Seq methods (Fig. 3). However, RNA-Seq tended to overestimate the amount of RNA compared to qPCR. Fig. 3 also illustrates changes in gene expression over time in the OURT-infected pigs. Several genes, including *CCL5*, *GPR56*, and *GZMA* maintained similar levels of expression between days 3 and 28. The qPCR results generated are included in Supplementary Table S9.

### Linking DEGs with cells and tissues

**Figure 4:**
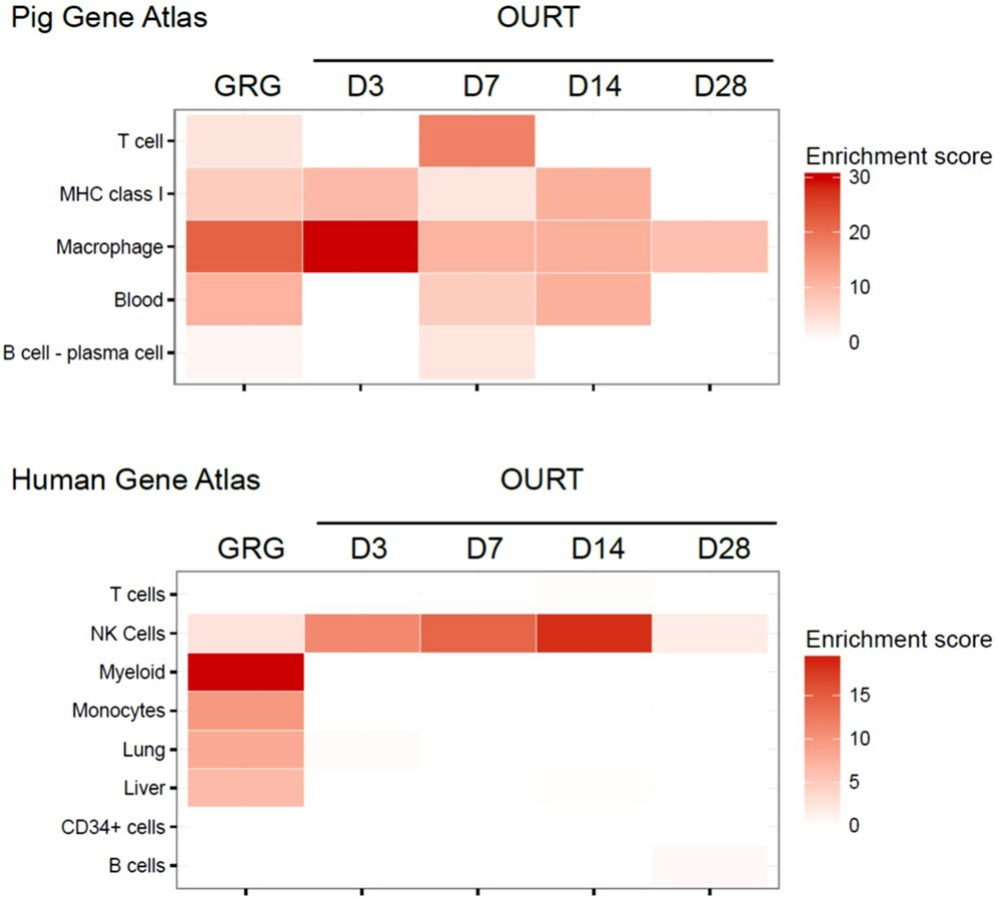
Cell and tissue types associated with upregulated DEGs following infection of pigs with GRG or OURT. Sets of upregulated DEGs were compared to the previously published gene expression atlases from humans^51^ and pigs^52^ to identify cells or tissues associated with the upregulated host genes. Results are shown as a heat map of enrichment scores for DEGs for selected cell or tissue types.

Pig and human gene atlases were used to link host gene expression with cells and tissues. The transcriptome profiles for cells following infection with both viruses were largely associated with immunity, consistent with upregulation of genes in monocytes, macrophages and lymphocytes. The GRG transcriptome profile largely reflected functions associated with myeloid cells and macrophages (Fig. 4). In addition, the Human Gene Atlas identified GRG pig gene expression profiles associated with lung and liver, which represent organ systems populated by relatively large numbers of ASFV-infected macrophages. For the OURT pigs, the human atlas showed increased gene profiles associated with NK cells, with maximum gene expression at 14 days after infection. This pattern supports the results from Table 4 and Fig. 2 showing increased NK cell-associated genes expression. The Pig Gene Atlas showed upregulated genes in OURT-infected pigs associated with immune function. Blood was the tissue associated with upregulated gene expression by the Pig Gene Atlas for OURT. The pig atlas identified macrophages as the primary cell type associated with gene expression, along with T cells; however, there was no specific designation for NK cells.

## Discussion

A next generation sequencing approach was used to characterize the transcriptome changes during infection with OURT, a low virulence strain of ASFV, and a highly pathogenic isolate, GRG. RNA-Seq was performed on RNA from whole blood, which contained a mixed population of white blood cells, including polymorphonuclear and mononuclear cell populations. Mononuclear cells are further divided into lymphocytes, which includes B cells, T cells and NK cells, as well as monocytes and macrophages. The presence of infected circulating monocytes in the GRG pigs allowed the detection and analysis of more than 110 ASFV genes and gene families (Table 1 and Supplementary Table S5). One ASFV gene that was highly expressed was *DP71L*, which codes for a protein that functions as a cofactor for host protein phosphatase 1 (PP1), a phosphatase responsible for dephosphorylating P-eIF2α. Homologs of DP71L include the herpes simplex virus (HSV) protein, ICP34.5^21^, and the host cell protein, GADD34^22^. In response to stress or infection, protein kinases, such as PKR and PERK, become activated and shutdown translation by phosphorylating eIF2α, a protein required for the initiation of translation. Dephosphorylation of P-eIF2a by PP1 and cofactors, such as GADD34, or a viral homolog, restores translation. The host gene *PPP1R15A*, which codes for GADD34, was highly upregulated following GRG infection (Table 2). Therefore, in concert with the expression of *DP71L*, upregulation of host *PPP1R15A* provides a redundant mechanism to ensure that eIF2α is maintained in a non-phosphorylated state during virus infection. For HSV, the replication of *ICP34.5* gene deletion mutants is highly attenuated, the result of the loss of PP1 cofactor activity and subsequent accumulation of P-eIF2α^23^. In contrast, ASFV *DP71L* deletion mutant viruses are not attenuated and P-eIF2α and translation levels remain the same as cells infected with wild-type virus^24^. It was suggested by the authors that other viral proteins may duplicate *DP71L* function^24^. However, the upregulation of host *PPP1R15A* explains why the loss of *DP71L* does not affect host cell translation during ASFV infection. After infection, the initial accumulation of P-eIF2α initiates the transcription of *ATF4*, which in turn upregulates the expression of unfolded protein response (UPR)-associated genes, including *PPP1R15A* and *CHOP* (an inducer of apoptosis). Therefore, the function of ASFV *DP71L* appears to be two-fold: the continuation of translation along with blocking the production of UPR-associated proteins, such as CHOP. A deeper analysis of the RNA-Seq data failed to reveal the upregulated expression of *ATF4* or *CHOP*, suggesting that upregulated *PPP1R15A* may be the result of an ATF4-independent mechanism, perhaps, through an ASFV-dependent pathway.

At the tissue level, human and pig gene atlases showed that the greatest number of genes upregulated in response to GRG infection were associated with cells of the myeloid lineage, which includes macrophages (Fig. 4). These data provide further support for the results presented in Tables 2 and 3 and support previous published reports describing increased numbers of circulating CD163-positive monocytes during acute ASFV infection^25^. Even though CD163 has been proposed as a candidate receptor for ASFV^26^, recent data show that CD163-knockout pigs support GRG infection and exhibit clinical signs consistent with acute infection^27^. The association of DEGs with the immune response was found in the results from the Human Atlas, including DEGs associated with NK and T cell function. Again, these results support the finding of upregulated genes associated with macrophages and NK function (Tables 2–4).

In support of the human and pig atlases, KEGG pathway analysis during OURT infection showed a significant number of DEGs associated with NK cell function (Table 4). Lieto *et al*. (2001) were the first to publish the observation of increased NK cell activity in pigs infected with the moderately pathogenic ASFV strain, ASFV/L60^28^. Based on clinical signs, infected pigs could be placed into one of two groups. Pigs that exhibited clinical signs consistent with a chronic infection showed no change in NK cell activity. In contrast, pigs exhibiting only mild clinical signs showed increased NK cell activity, which peaked at seven days after infection and then declined to near background levels by 35 days. In a similar manner, the OURT pigs in our study showed elevated NK-associated gene expression between days 3 and 14 after infection (Fig. 4), thus mimicking the response of the pig to a mild infection. Compared to the OURT pigs, NK cell-associated gene expression was only slightly elevated in the GRG pigs. The role of NK cells in the control of ASFV infection remains unclear and deserves further study. KEGG pathway analysis of DEGs from GRG-infected pigs identified genes associated with the RLR signaling pathway. This pathway involves the detection of viral infection by RLRs and the subsequent activation of interferons and proinflammatory cytokines^29^. While this finding is consistent with the DEGs observed following GRG infection (Table 2, Supplementary Table S6), the RLR pathway is associated with RNA virus infection. This finding for a DNA virus will require experimental validation before its significance can be determined.

Using a panel of gene-specific PCR primers, Fishbourne *et al*. described the upregulation of several macrophage-associated chemokine and chemokine receptor genes in whole blood RNA from pigs infected with OURT or the virulent strain, Benin 97/1^19^. Genes described as upregulated included *CCL2, CCL3, CCL4, CCL5, CCR1, CCR5, CCL3L1, CXCL10, CCR1, CCR5, CCR9, CXCR4*. We also detected the differential expression of the same genes, with the additional observation of upregulation of *CX3CR1* (Supplementary Table S6).

RNA-Seq revealed only a limited number of miRNA genes upregulated during ASFV infection, including *miR-122, miR-138, miR-181A, miR-199A-2*, and *miR-1296* (Tables 2 and 3, Supplementary Table S6). There are no previous studies that can provide insight into miRNAs during ASFV infection. The current observation departs from results for other macrophage-tropic virus. Since we did not specifically target miRNAs during the RNA purification step, the most likely explanation is the result of an under-representation due to the RNA purification method used in this work. Potential roles for miRNAs in ASFV infection have not been investigated, but like other viral infections, miRNAs are likely involved in the regulation of virus replication and host responses. For example, circulating levels of miR-122 are upregulated in response to liver damage caused by hepatitis C infection^20^. The detection of miR-122 in the circulation of GRG pigs (Table 2) could be the result of liver damage cause by the ASFV infection of hepatic-specific macrophages, such as Kupffer’s cells^30^. Interestingly, another liver-associated miRNA gene, *miR-199* was upregulated at 28 days after infection with OURT. Other miRNAs, such as miR-181A, are essential for NK cell ontogenesis and T lymphocyte development^31^. Since it appears that the number of miRNAs produced during ASFV infection may be limited, the detection of unique ASF-specific miRNAs could provide a diagnostic tool to identify sero-negative infected animals.

Overall, a large percentage of genes were identified as downregulated relative to the day 0 control. As discussed above, the most highly downregulated genes could not be linked to virus infection, inflammation or lymphocyte activation. There may be multiple sources of downregulation of DEGs. For example, over the 28 day period of the OURT study, certain developmental-related genes may become downregulated, a result of the pig becoming older. Another source of downregulation may be the result of a change in cell subpopulations because of infection. One limitation of this study was that we did not perform a phenotypic analysis of cell populations. Increase in certain cytokines may also influence DEGs. As examples, the two most highly downregulated genes in the GRG pigs were *GCNT4*, which codes for a protein glycosylation enzyme, glucosaminyl (N-acetyl) transferase and *GAMT*, which encodes guanidinoacetate methyl transferase. *GCNT4* expression is associated with breast cancer outcomes^32^ and the downregulation of *GAMT* is associated with cerebral creatine deficiency syndrome^33^. The role of gene downregulation during ASF infection deserves further study.

Weaner piglets were used in this study. Although pigs of this age may not be considered immunologically fully mature, ASFV is known to infect pigs of all ages, and the use of younger pigs for ASFV experimental infection has been used in previous studies^13, 14^. Recent findings by Post *et al*., which compared 12 vs 18 week old domestic pigs, revealed differences in susceptibility to a moderately virulent strain as well as differences in early T cell and IL-10 responses^34^. However, their results differed from an early study that showed no mortalities in 12 week old pigs^35^. Determining whether there are age-related differences in the immune transcriptome following ASFV infection with these virus strains would need to be addressed by a separate study.

Pigs in the OURT group were found to be co-infected with porcine circovirus type-2 (PCV-2), based on PCR and serological testing of pre- and post-challenge blood samples. PCV-2 is ubiquitous in pig populations and concurrent infections with other pathogens, such as porcine parvovirus and porcine reproductive and respiratory syndrome virus, are known to trigger PCV-associated disease (PCVAD)^36^. Although we found no evidence of PCVAD, this does not preclude the OURT pigs from having subclinical PCV-2 infection. In sub-clinical infections, PCV-2 is thought to be internalized within immune cells, such as dendritic cells and monocytes, with minimal or no replication or alteration to the immune function of these cells^37^. Samples from two of the three pigs analyzed in the OURT group were negative for PCV-2 DNA at day 7 PI; therefore, it may be concluded that the transcriptome changes observed in these samples are in response to infection with ASFV OURT. Furthermore, cytokine responses previously reported following PCV-2 subclinical infection^38, 39^ were not observed in the OURT pigs at any time point analyzed (Supplementary Table S6). This suggests that the host responses we observed were not influenced by PCV2 infection, but we cannot rule this out.

The application of RNA-Seq for the analysis of gene expression during ASFV infection supports much of the previous data describing host responses to ASFV infection and further extends our knowledge of the virus-host relationship. RNA-Seq approaches have the potential to provide valuable information on new vaccine strategies that stimulate protective immunity. The use of more targeted methods for small RNA sequencing are also expected to further our understanding of the miRNA response to ASFV infection and the influence of individual miRNAs on ASFV replication.

## Methods

### Ethics Statement

Animal work was approved by the CSIRO Australian Animal Health Laboratory (AAHL) Animal Ethics Committee (permit number 1673). All procedures were conducted per the guidelines of the National Health and Medical Research Council as described in the Australian code for the care and use of animals for scientific purposes, 8^th^ edition (2013).

### Viruses used in this study

OURT and GRG isolates have been described previously^10, 12^. The Vero cell-adapted strain, BA71V^40^, was used as antigen for the IFA assay. All viruses were generously provided by Dr. Linda Dixon (Pirbright Institute, United Kingdom). All *in vitro* and *in vivo* work involving live ASFV was conducted within biosafety level 3 facilities at the AAHL. GRG and OURT were cultured in primary porcine bone marrow (PBM) cells using RPMI media supplemented with glutamine, HEPES, penicillin and streptomycin, Fungizone and 12.5% fetal calf serum (FCS, Serana)^41, 42^. After 4 days incubation, inoculum was removed, cells were washed, then fixed with cold acetone/methanol for 30 min at room temperature. Cells were washed three times with PBS and incubated for 1 hr at 37 °C with an in-house rabbit anti-ASFV p32 polyclonal antibody diluted in PBS with 1% BSA and 0.005% Evans Blue. Following three washes, Alexa Fluor 488 protein A conjugate (ThermoFisher) was added to each well and incubated for 30 min at 37 °C. Following a final wash step, fluorescence was visualized with an IX71 Olympus inverted fluorescence microscope. TCID_50_ was calculated according to the method of Reed and Muench^43^.

### Experimental infection of pigs

Groups of 5–6 weeks old Landrace cross domestic pigs were used for each infection trial. After a period of approximately one week acclimatisation in the animal rooms, six pigs were inoculated with OURT, while four pigs were inoculated with GRG. Prior to challenge, serum and whole blood were collected from each pig to confirm that animals were negative for antibody to ASFV and viral DNA using the INGEZIM PPA Compac R.11.PPA.K3 blocking ELISA, and an ASFV-specific PCR^44^. Pigs were also screened for influenza A virus and porcine circovirus types 1 and 2 using in-house PCR and serology tests. A dose of 10^6^ TCID_50_ in 4.0 mL was administered via the oro-nasal route to simulate natural infection. The inoculum was administered to anaesthetized pigs held in dorsal recumbency by slow dropwise instillation of 1.0 mL into each nostril and 2.0 mL into the oral cavity. This method minimized ingestion following administration. Back-titrations were performed to confirm the dose. Pigs were monitored daily then twice daily or more following the first appearance of clinical signs. A quantitative clinical scoring system was employed in combination with qualitative endpoint criteria (Supplementary Table S10)^45^. Pre-determined humane endpoints included pigs displaying moderate disease signs progressing towards severe disease, moderate disease signs persisting for more than three consecutive days, or severe disease signs. Pigs that reached the humane endpoint were sedated followed by a barbiturate overdose.

For OURT-inoculated pigs, blood and swabs (oral, nasal, faecal) were collected prior to inoculation then on day 3, 7, 14, 21 and 28 PI. For GRG-inoculated pigs, blood and swabs were also collected prior to inoculation, and at the time of euthanasia (7 and 10 days PI); additional swabs were collected on days 3, 5, 7, 9 PI. Blood was collected in serum clotting, EDTA and Tempus Blood RNA tubes. Swabs were placed into PBS containing antibiotics. Tissues samples from the liver, spleen, kidney, lung, tonsil, and lymph nodes were collected and homogenized with 1-mm silicon carbide beads (BioSpec Products) twice for 20 sec in a FastPrep24 tissue homogenizer for virus titration and PCR. Comparative tissue samples were also collected in 10% formalin and RNAlater for histopathological and RNA-Seq analyses, respectively.

### Indirect fluorescent antibody for measurement of ASFV antibodies

Serum was heat-inactivated at 60 °C for 1 hr prior to serological testing. Vero cells (ATCC, CCL-81) were cultured in EMEM supplemented growth media in 96-well plates and then infected with ASFV strain BA71V at a MOI of 0.02. After 1 hr incubation, the inoculum was removed and maintenance media was added. Plates were incubated for 48 hrs at 37 °C and cell monolayers were washed and fixed. Serum samples (pre-diluted 1:5) were serially diluted 4-fold in PBS with 1% BSA and 0.005% Evans Blue, incubated for 1 hr at 37 °C then washed three times. Antibody bound to infected cells was detected by Alexa Fluor 488 protein A conjugate and immunofluorescence.

### Histology and immunohistochemistry

Following fixation in 10% neutral buffered formalin, tissues were sectioned and processed for haematoxylin-eosin staining and immunohistochemistry. Sections were rehydrated with water prior to antigen retrieval in Dako PT link with EnVision Flex target retrieval solution, high pH for 20 min at 97 °C. The slides were then washed for 5 min prior to staining using a Dako Autostainer Link 48. The sections were treated with 3% hydrogen peroxide for 10 min to quench any endogenous peroxidases and in-house rabbit polyclonal anti-ASFV p32 and p72 were applied for 45 min. EnVision Flex HRP was applied as the secondary antibody to each section for 20 min and AEC+ high sensitivity substrate chromogen was added for a further 10 min. Sections were counterstained with Lillie-Mayer haematoxylin (Australian Biostain) before mounting with Faramount aqueous mounting medium.

### Isolation of total RNA from blood

Viral DNA for ASFV PCR testing was extracted from blood, swabs and tissues using the Ambion MagMAX^TM^−96 Viral Isolation Kit and the MagMAX^TM^ Express-96 Magnetic Particle Processor. Blood samples for RNA-Seq analysis were extracted using the Ambion MagMAX^TM^ for Stabilized Blood Tubes RNA Isolation Kit, up to completion of the DNase step. Briefly, 130 µL of each DNase-treated sample was mixed with 50 µL of Binding Solution and 20 µL of magnetic bead preparation, then added to 200 µL of isopropanol, mixed again, then extracted. All elution volumes were 80 µL.

### ASFV PCR

PCR was performed on whole blood, swabs and tissue specimen extracts using a modification of the assay described by King *et al*.^44^. PCR assays used 5 μL of DNA template, sense and antisense primers (900 nM) and TaqMan® probe (250 nM), and AgPath-ID one-step RT-PCR reagents (ThermoFisher) in a 25 µL reaction volume. Thermocycling conditions were 45 °C 10 min, 95 °C 10 min, and 45 cycles of 95 °C 15 sec, 60 °C 45 sec.

### RNA sequencing

A total volume of 80 µL of RNA extracted from each sample was shipped to Lawrence Livermore National Laboratory. Agilent Bioanalyzer analysis was performed on all samples using the Agilent RNA 6000 Pico kit to check the integrity of RNA. RNA samples were submitted to UC Berkeley Genome Center for RNA-Seq analysis on Illumina HiSeq. 2000 using the 50 bp single end reads sequencing method. Library preparation was done using the PrepX RNA-Seq sample and library preparation kits with Ribo-Zero depletion to remove unwanted rRNA present in the samples^46^ (Wafergen Biosystems). RNA from three randomly selected pigs (numbers 7, 11, 12) infected with OURT and three randomly selected pigs (numbers 13, 14 and 16) infected with GRG were sequenced.

### Bioinformatics analysis

The pig genome assembly was downloaded from the UCSC Browser, Assembly 10.2 (8/2011). Approximately 5,000 annotated genes were obtained from GenBank, and around 30,000 genes were retrieved from Ensembl, version 10.2.82. Gene annotation was determined using the mapped RNA locus that overlaps with the annotated gene from the identified strand. Pig genes were then mapped to the human orthologs using OrthoDB version 8^47^ and corresponding Ensembl gene identifiers. Gene name assignments were given using *Sus scrofa domesticus* genome annotation when possible, otherwise the human ortholog gene name was used.

Lists of differentially expressed genes (DEGs) were prepared using the tuxedo pipeline^48^. First, the reads were mapped to the pig reference, then the transcripts were assembled to measure abundance on a per gene loci basis. Gene expression was measured as Fragments Per Kilobase per Million mapped fragments (FPKM), which counts the number of reads mapped to each transcript overlapping each gene locus. The read counts were normalized by transcript length and summed across all transcripts associated with the gene locus. In addition, the read count value was normalized by the total read count to support comparisons across samples with differing numbers of sequenced reads. Transcripts and genes were compared using Cuffdiff^49^ across samples to link the shared loci, and finally a model for abundance counts for each transcribed gene along with variance were built to identify genes with abundance counts significantly above or below the expected value. The false discovery rate adjusted p-values (q-values) were taken to measure significant differential gene expression. These values evaluated the distribution of p-values across all tested genes to estimate a significance threshold after adjusting for multiple hypotheses testing of the multiple gene loci. To focus on genes with the greatest consistent differential gene expression values, genes were sorted and ranked by their q-values. Genes with the lowest q-values were selected for further examination.

Reads were also mapped to the ASFV reference Georgia 2007/1 complete genome (Genbank accession number: FR682468.1). The average coverage depth for each gene was calculated and ranked. Read counts were normalized by the total number of reads in each sample for cross sample comparison. To consider the potential influence of “read through” in transcripts, expression values for the top expressed genes were compared with its immediate gene neighbors. The ratio of the FPKM value between the target gene and neighbor with minimum value was taken to identify cases where high expression in the neighboring gene may explain expression in the target gene. A cutoff of 2 was chosen to flag genes where the target gene’s FPKM value was less than double the neighboring gene. An additional requirement was made that the condition was observed in more than one pig.

### Gene Ontology and mapping to KEGG pathways

Differentially expressed transcripts were annotated using the Database of Annotation, Visualization, and Integrated Discovery (DAVID) version 6.7^50^. The most significant DEGs were mapped to KEGG pathways and gene identifications were retrieved. The DAVID gene enrichment tool was used to report KEGG pathways and GO terms that were overrepresented within the top DEGs. P values were calculated using Fisher exact test based on the fraction of genes that mapped to a specific pathway as compared to the number of background genes associated with the pathway.

To identify the cell and tissue sources of upregulated DEGs, we compared the expression profile of upregulated DEGs to databases of curated protein-encoding transcriptomes, including the Human Gene Atlas and Pig Gene Atlas^51, 52^. This analysis was performed in conjunction with the gene set enrichment tool, Enrichr^53, 54^. The enrichment scores for specific cell or tissue types were determined using the combined score method^53^.

### QPCR of DEGs

QPCR was performed on several of the highly-upregulated genes to confirm the RNA-Seq results. Reaction parameters were identical for all genes. Briefly, 1 μg of total RNA was reverse transcribed using iScript Reverse Transcription Supermix (Bio-Rad) with oligo-dT and random primers. Triplicate SYBR green real-time PCR reactions were performed in a 20 μL reaction, containing 1X iTaq SYBR Green Supermix, 1X PrimePCR SYBR Green Assay forward and reverse primer, and 1 μL cDNA template diluted to 50 μL. Cycling parameters were 95 °C for 20 s, 40 cycles of 95 °C for 3 s and 60 °C for 30 s, followed by melt curve analysis. Differential expression was calculated relative to uninfected D0, normalized to the β-2-microglobulin gene.

### Data availability

All data generated and analyzed from this study are included in this published article (and its Supplementary files). The raw RNA-Seq data has been submitted to NCBI Short Read Archive (SRA) under SRP108916 and Bioproject PRJNA389819.

## Electronic supplementary material

Supplementary Info

Table S5

Table S6

Table S8

Table S9
