# Supplementary material for: Gene expression analysis of whole blood RNA from pigs infected with low and high pathogenic African swine fever viruses
Source: Sci Rep. 2017 Aug 31;7:10115. doi: 10.1038/s41598-017-10186-4 (PMC5579198; doi:10.1038/s41598-017-10186-4)
Supplement: Supplementary file 1 — Supplementary Info [file 41598_2017_10186_MOESM1_ESM.pdf]

Supplementary Information

Gene expression analysis of whole blood RNA from pigs infected with low and high pathogenic African swine fever viruses

Crystal Jaing[1,*], Raymond R.R. Rowland[2], Jonathan E. Allen[3], Andrea Certoma[4], James B. Thissen[1], John Bingham[4], Brenton Rowe[4], John R. White[4], James W. Wynne[4], Dayna Johnson[4], Natasha N. Gaudreault[2], David T. Williams[4]

[1]Physical & Life Sciences Directorate, Lawrence Livermore National Laboratory, Livermore, California, United States of America
[2]Department of Diagnostic Medicine and Pathobiology, Kansas State University, Manhattan, Kansas, United States of America
[3]Computation Directorate, Lawrence Livermore National Laboratory, Livermore, California, United States of America
[4]CSIRO Australian Animal Health Laboratories, Geelong, Victoria, Australia
*corresponding author, jaing2@llnl.gov

Supplementary Fig. S1. Individual clinical scores for OURT-infected (a) and GRG - infected pigs (b).

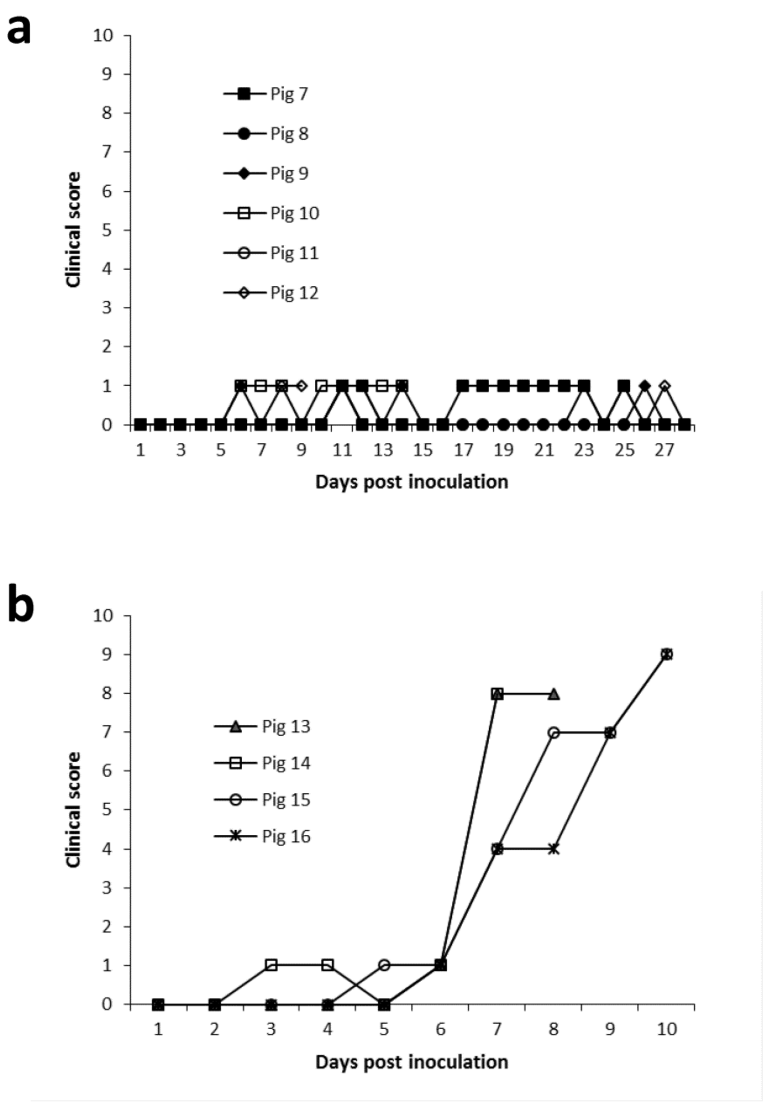

Supplementary Fig. S2. African swine fever viral antigen in tissues infected with the highly virulent Georgia 2007/1 strain. (A) Spleen (pig 13), (B) lung (pig 14), (C) tonsil (pig 14), showing p30 viral antigen in single cells, probably mainly macrophages, within tissue parenchyma. (D) Liver (pig 16), showing p72 viral antigen within hepatocytes, including cytoplasmic inclusion bodies (arrows) corresponding to viral factories. Immunohistochemistry used AEC chromogen (brick red colour) and haematoxylin counterstain.

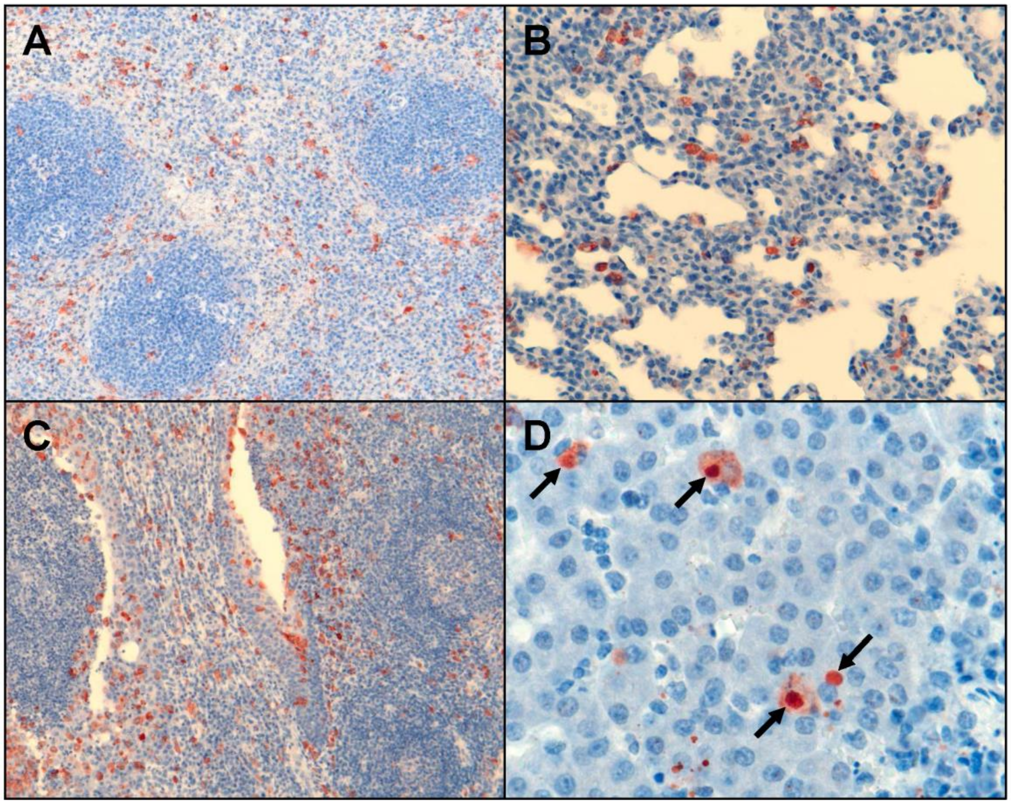

Supplementary Table S1. ASFV DNA in oral, nasal and fecal swabs of GRG pigs measured by real-time PCR.

	Days post inoculation						
Pig and swab type	0	1	3	5	7	9	10
Pig 13							
Oral swab	No C_t[a]	No C_t	No C_t	No C_t	37.3		
Nasal swab	No C_t	No C_t	NT[b]	40.7	36.6		
Fecal swab	No C_t	No C_t	NT	No C_t	No C_t		
Pig 14							
Oral swab	No C_t	No C_t	NT	No C_t	29.7		
Nasal swab	No C_t	No C_t	No C_t	30.2	23.8		
Fecal swab	No C_t	No C_t	No C_t	32.0	27.1		
Pig 15							
Oral swab	No C_t	No C_t	No C_t	No C_t	31.2	27.9	30.3
Nasal swab	No C_t	No C_t	37.6	38.4	23.3	NT	25.9
Fecal swab	No C_t	No C_t	No C_t	No C_t	29.8	NT	29.9
Pig 16							
Oral swab	No C_t	No C_t	No C_t	No C_t	30.5	30.4	30.8
Nasal swab	No C_t	No C_t	No C_t	36.4	26.7	25.9	26.6
Fecal swab	No C_t	No C_t	No C_t	NT	30.6	33.6	29.3

[a] The measurement of ASFV nucleic acid was measured by real-time PCR; Ct, cycle threshold.
[b]NT, not tested.

Supplementary Table S2. ASFV in whole blood and tissues of GRG pigs measured by real-time PCR.

Pig no.	Day	Ct value (log_{10} $TCID_{50}$/(mL of blood or gram of tissue))[a]								
		Blood	Liver	Spleen	Kidney	Lung	Tonsil	Submandib lymph node	Hepatic lymph node	Renal lymph node
13	8	21.7 (9.5)	20.9 (NT[b])	16.7 (8.5)	26.7 (NT)	20.2 (NT)	29.8 (NT)	25.0 (5.2)	23.8 (6.0)	25.7 (6.3)
14	7	19.9 (10.0)	20.9 (NT)	17.8 (8.5)	26.3 (NT)	21.3 (NT)	26.5 (NT)	20.7 (8.3)	21.0 (8.5)	22.8 (8.2)
15	7	23.6 (9.8)	17.2 (NT)	20.4 (8.5)	22.4 (NT)	21.0 (NT)	19.8 (NT)	19.7 (7.8)	19.2 (8.3)	19.3 (8.5)
16	10	23.3 (10.0)	16.6 (NT)	19.0 (8.0)	23.3 (NT)	20.8 (NT)	21.0 (NT)	19.3 (8.8)	19.8 (8.8)	20.4 (8.3)

[a] The measurement of ASFV nucleic acid was measured by real-time PCR. The results for virus isolation are shown in parentheses.

[b]NT, not tested.

Supplementary Table S3. Total number of reads and mapped reads from RNAseq of blood samples from ASFV OURT and GRG infected pigs. Total reads obtained is number of sequencer reads compared against the pig reference genome. Reads Mapped Once is the number of reads that were mapped to one loci in the pig reference genome. Fraction of reads mapped once is the number of reads mapped once divided by the total reads obtained. The overall alignment rate reports the fraction of all reads mapped to the reference genome, both reads that map to one genomic loci and reads that map to more than one genomic loci.

SAMPLE NO	PIG	Experimental day	Total Reads	Reads Mapped Once	Fraction of reads mapped once	Overall alignment rate
OURT						
14-01350-001	7	D0	37766686	26268754	69.56%	77.02%
14-01350-037	7	D3	40296019	29501635	73.21%	82.02%
14-01350-073	7	D7	52796074	37112590	70.29%	78.35%
14-01350-109	7	D14	38376116	27947617	72.83%	80.84%
14-01350-181	7	D28	44664842	31606145	70.76%	79.85%
14-01350-025	11	D0	52812050	34167405	64.70%	74.32%
14-01350-061	11	D3	42233351	29220177	69.19%	78.54%
14-01350-097	11	D7	36771587	25744407	70.01%	80.18%
14-01350-133	11	D14	41310046	29572707	71.59%	80.44%
14-01350-169	11	D21	42294925	30724131	72.64%	83.19%
14-01350-205	11	D28	40057060	27595845	68.89%	78.11%
14-01350-031	12	D0	43956125	30341908	69.03%	78.84%
14-01350-067	12	D3	44812580	30708459	68.53%	78.89%
14-01350-103	12	D7	58108329	36756649	63.26%	73.99%
14-01350-139	12	D14	43037924	30066089	69.86%	81.52%
14-01350-175	12	D21	36098672	26303234	72.86%	81.50%
14-01350-211	12	D28	39313539	28784655	73.22%	82.73%
Average			43,217,996	30,142,495	70.03%	79.43%
GRG						
14-03987-001	13	D0	35587260	21041353	59.13%	88.45%
14-03987-100	13	ED8	60894648	34770809	57.10%	86.92%
14-03987-007	14	D0	32141100	17003090	52.90%	89.13%
14-03987-110	14	ED7	39099846	21984959	56.23%	84.67%
14-03987-019	16	D0	26603260	16324365	61.36%	87.74%
14-03987-130	16	ED10	32801024	19435533	59.25%	85.86%
Average			37,854,523	21,760,018	57.66%	87.13%

Supplementary Table S4. Differentially expressed genes (DEGs) during infection with GRG or OURT infection[a].

Comparison		Number of DEGs	Number Up-regulated	Number Down-regulated	q value[c]
GRG: D0 vs ED[b]					
Pig 13		312 (132)	166 (51)	146 (81)	
Pig 14		316 (148)	171 (67)	145 (81)	
Pig 16		316 (154)	170 (50)	146 (104)	
	Total	395 (186)	217 (76)	178 (110)	0.0023
OURT: D0 vs D3					
Pig 7		27 (3)	18 (3)	9 (0)	
Pig 11		25 (5)	17 (3)	8 (2)	
Pig 12		26 (6)	18 (3)	8 (3)	
	Total	51 (10)	28 (6)	23 (4)	0.0057
OURT: D0 vs D7					
Pig 7		142 (13)	41 (12)	101 (1)	
Pig 11		141 (14)	41 (9)	100 (5)	
Pig 12		143 (18)	42 (14)	101 (4)	
	Total	181 (25)	52 (18)	129 (7)	0.0081
OURT: D0 vs D14					
Pig 7		37 (12)	29 (10)	8 (2)	
Pig 11		38 (8)	29 (5)	9 (3)	
Pig 12		35 (14)	26 (110	9 (3)	
	Total	52 (22)	36 (16)	16 (6)	0.0168
OURT: D0 vs D28					
Pig 7		462 (19)	57 (5)	405 (14)	
Pig 11		450 (2)	56 (2)	394 (0)	
Pig 12		461 (14)	59 (4)	402 (10)	
	Total	490 (22)	74 (8)	416 (14)	0.0056

[a]The numbers in parentheses show genes that were classified as "highly differentially expressed" (expression level >2log$_2$).

[b]ED, end date for three pigs sacrificed at 7, 8 and 10 days after infection.

[c]Top ranked false discovery rate adjusted p-value for gene expression values in each pairwise comparison category.

Supplementary Table S7. List of Kegg pathway analysis results from GRG and OURT pigs

**Up regulated pathways**		
Sample type	KEGG PATHWAY ID	KEGG PATHWAY DESCRIPTION
Both	hsa00330	Arginine and proline metabolism
Both	hsa04010	MAPK signaling pathway
Both	hsa04020	Calcium signaling pathway
Both	hsa04060	Cytokine-cytokine receptor interaction
Both	hsa04062	Chemokine signaling pathway
Both	hsa04080	Neuroactive ligand-receptor interaction
Both	hsa04144	Endocytosis
Both	hsa04210	Apoptosis
Both	hsa04630	Jak-STAT signaling pathway
Both	hsa04650	Natural killer cell mediated cytotoxicity
Both	hsa04722	Neurotrophin signaling pathway
Both	hsa04810	Regulation of actin cytoskeleton
Both	hsa04940	Type I diabetes mellitus
Both	hsa05130	Pathogenic Escherichia coli infection
Both	hsa05200	Pathways in cancer
Both	hsa05222	Small cell lung cancer
Both	hsa05320	Autoimmune thyroid disease
Both	hsa05330	Allograft rejection
Both	hsa05332	Graft-versus-host disease
Both	hsa05416	Viral myocarditis
GRG	hsa00790	Folate biosynthesis
GRG	hsa04360	Axon guidance
OURT D7	hsa00071	Fatty acid metabolism
OURT D7	hsa00430	Taurine and hypotaurine metabolism
OURT D7	hsa00480	Glutathione metabolism
OURT D7	hsa02010	ABC transporters
OURT D7	hsa03018	RNA degradation
OURT D7	hsa03030	DNA replication
OURT D7	hsa03040	Spliceosome
OURT D7	hsa03320	PPAR signaling pathway
OURT D7	hsa04110	Cell cycle
OURT D7	hsa04114	Oocyte meiosis
OURT D7	hsa04115	p53 signaling pathway
OURT D7	hsa04120	Ubiquitin mediated proteolysis
OURT D7	hsa04142	Lysosome
OURT D7	hsa04150	mTOR signaling pathway

OURT D7	hsa04260	Cardiac muscle contraction
OURT D7	hsa04610	Complement and coagulation cascades
OURT D7	hsa04612	Antigen processing and presentation
OURT D7	hsa04620	Toll-like receptor signaling pathway
OURT D7	hsa04621	NOD-like receptor signaling pathway
OURT D7	hsa04622	RIG-I-like receptor signaling pathway
OURT D7	hsa04623	Cytosolic DNA-sensing pathway
OURT D7	hsa04660	T cell receptor signaling pathway
OURT D7	hsa04662	B cell receptor signaling pathway
OURT D7	hsa04710	Circadian rhythm
OURT D7	hsa04720	Long-term potentiation
OURT D7	hsa04910	Insulin signaling pathway
OURT D7	hsa04914	Progesterone-mediated oocyte maturation
OURT D7	hsa04920	Adipocytokine signaling pathway
OURT D7	hsa04960	Aldosterone-regulated sodium reabsorption
OURT D7	hsa05014	Amyotrophic lateral sclerosis (ALS)
OURT D7	hsa05020	Prion diseases
OURT D7	hsa05110	Vibrio cholerae infection
OURT D7	hsa05120	Epithelial cell signaling in Helicobacter pylori infection
OURT D7	hsa05215	Prostate cancer
OURT D7	hsa05220	Chronic myeloid leukemia
OURT D7	hsa05310	Asthma
**Downregulated pathways**		
Both	hsa00330	Arginine and proline metabolism
Both	hsa00500	Starch and sucrose metabolism
Both	hsa00520	Amino sugar and nucleotide sugar metabolism
Both	hsa00910	Nitrogen metabolism
Both	hsa04110	Cell cycle
Both	hsa04670	Leukocyte transendothelial migration
Both	hsa05200	Pathways in cancer
Both	hsa05220	Chronic myeloid leukemia
GRG	hsa00010	Glycolysis / Gluconeogenesis
GRG	hsa00051	Fructose and mannose metabolism
GRG	hsa00052	Galactose metabolism
GRG	hsa00250	Alanine
GRG	hsa00310	Lysine degradation
GRG	hsa00471	D-Glutamine and D-glutamate metabolism
GRG	hsa00480	Glutathione metabolism

GRG	hsa00900	Terpenoid backbone biosynthesis
GRG	hsa00980	Metabolism of xenobiotics by cytochrome P450
GRG	hsa00982	Drug metabolism
GRG	hsa03320	PPAR signaling pathway
GRG	hsa04010	MAPK signaling pathway
GRG	hsa04060	Cytokine-cytokine receptor interaction
GRG	hsa04062	Chemokine signaling pathway
GRG	hsa04115	p53 signaling pathway
GRG	hsa04120	Ubiquitin mediated proteolysis
GRG	hsa04144	Endocytosis
GRG	hsa04210	Apoptosis
GRG	hsa04610	Complement and coagulation cascades
GRG	hsa04630	Jak-STAT signaling pathway
GRG	hsa04666	Fc gamma R-mediated phagocytosis
GRG	hsa04910	Insulin signaling pathway
GRG	hsa04916	Melanogenesis
GRG	hsa04920	Adipocytokine signaling pathway
GRG	hsa04930	Type II diabetes mellitus
GRG	hsa05016	Huntington's disease
GRG	hsa05214	Glioma
GRG	hsa05215	Prostate cancer
GRG	hsa05218	Melanoma
GRG	hsa05219	Bladder cancer
GRG	hsa05221	Acute myeloid leukemia
OURT D7	hsa00190	Oxidative phosphorylation
OURT D7	hsa00260	Glycine
OURT D7	hsa00860	Porphyrin and chlorophyll metabolism
OURT D7	hsa00903	Limonene and pinene degradation
OURT D7	hsa01040	Biosynthesis of unsaturated fatty acids
OURT D7	hsa03010	Ribosome
OURT D7	hsa04114	Oocyte meiosis
OURT D7	hsa04140	Regulation of autophagy
OURT D7	hsa04270	Vascular smooth muscle contraction
OURT D7	hsa04310	Wnt signaling pathway
OURT D7	hsa04350	TGF-beta signaling pathway
OURT D7	hsa04360	Axon guidance
OURT D7	hsa04510	Focal adhesion
OURT D7	hsa04512	ECM-receptor interaction
OURT D7	hsa04514	Cell adhesion molecules (CAMs)

OURT D7	hsa04520	Adherens junction
OURT D7	hsa04530	Tight junction
OURT D7	hsa04540	Gap junction
OURT D7	hsa04640	Hematopoietic cell lineage
OURT D7	hsa04662	B cell receptor signaling pathway
OURT D7	hsa04810	Regulation of actin cytoskeleton
OURT D7	hsa05110	Vibrio cholerae infection
OURT D7	hsa05120	Epithelial cell signaling in Helicobacter pylori infection
OURT D7	hsa05130	Pathogenic Escherichia coli infection
OURT D7	hsa05210	Colorectal cancer
OURT D7	hsa05212	Pancreatic cancer
OURT D7	hsa05222	Small cell lung cancer
OURT D7	hsa05322	Systemic lupus erythematosus
OURT D7	hsa05340	Primary immunodeficiency
OURT D7	hsa05410	Hypertrophic cardiomyopathy (HCM)
OURT D7	hsa05412	Arrhythmogenic right ventricular cardiomyopathy (ARVC)
OURT D7	hsa05414	Dilated cardiomyopathy

Supplementary Table S10: Clinical signs and scoring system for pigs infected with African swine fever virus[a].

**Disease Severity**	**Score**	**Clinical signs**
None	0	None
Mild	1	Fever (rectal temp) >40.5°C<br>Reduced appetite, unresponsiveness to disturbance<br>Haemorrhages on the skin but limited in distribution<br>Mild cough and mild diarrhoea
Moderate	2	Fever >40.5°C<br>Inappetence or lethargy<br>More extensive skin haemorrhages<br>Cough or increased respiratory effort<br>Diarrhoea or vomiting<br>Lameness
Severe	3	Fever >41°C for more than 2 days<br>Depression, inappetence, poor responsiveness to disturbance<br>Skin haemorrhages and/or necrotic lesions covering 25% or more of body<br>Severe respiratory effort<br>Neurological signs such as tremors, paresis or seizure

[a]Based on Galindo-Cardiel et al. (2013); a combined score of 0-9 resulted in no action, with increased monitoring performed following progression to moderate disease. When more than one parameter was scored 3, all 3s were automatically converted to 4. Pigs were euthanized if scores of 10-18 were observed or if clinical signs met qualitative endpoints. Pre-determined humane endpoints included pigs displaying moderate disease signs progressing towards severe disease, moderate disease signs persisting for more than three consecutive days, or severe disease signs.
